# Miniaturized Antimicrobial Susceptibility Test by Combining Concentration Gradient Generation and Rapid Cell Culturing

**DOI:** 10.3390/antibiotics4040455

**Published:** 2015-10-29

**Authors:** Samuel C. Kim, Stefano Cestellos-Blanco, Keisuke Inoue, Richard N. Zare

**Affiliations:** 1 Department of Chemistry, Stanford University, Stanford, CA 94305, USA; E-Mails: samuel.kim2@ucsf.edu (S.C.K.); keisuke.inoue@takeda.com (K.I.); 2 Department of Chemical Engineering, Stanford University, Stanford, CA 94305, USA; E-Mail: scestell@stanford.edu

**Keywords:** antibiotics, AST, microfluidics, microdevice, lab-on-a-chip, MIC

## Abstract

Effective treatment of bacterial infection relies on timely diagnosis and proper prescription of antibiotic drugs. The antimicrobial susceptibility test (AST) is one of the most crucial experimental procedures, providing the baseline information for choosing effective antibiotic agents and their dosages. Conventional methods, however, require long incubation times or significant instrumentation costs to obtain test results. We propose a lab-on-a-chip approach to perform AST in a simple, economic, and rapid manner. Our assay platform miniaturizes the standard broth microdilution method on a microfluidic device (20 × 20 mm) that generates an antibiotic concentration gradient and delivers antibiotic-containing culture media to eight 30-nL chambers for cell culture. When tested with 20 μL samples of a model bacterial strain (*E. coli* ATCC 25922) treated with ampicillin or streptomycin, our method allows for the determination of minimum inhibitory concentrations consistent with the microdilution test in three hours, which is almost a factor of ten more rapid than the standard method.

## 1. Introduction

Effective treatment of bacterial infections relies on timely diagnosis and proper prescription of antibiotic drugs. It is customary to carry out the antimicrobial susceptibility test (AST) on isolated patient samples or common laboratory strains to determine best treatment options. AST provides the minimal inhibitory concentration (MIC) of an antibiotic drug for a bacterial strain, which is the lowest dose that will suppress bacteria from proliferating. The MIC profile of the infectious bacteria of interest can guide clinicians to prescribe an effective concentration of drug and prevent bacteria from developing resistance before being eradicated. In view of the rapid growth rate of bacteria in the human body, quick determination of AST results can be highly beneficial especially in the case of treating unknown bacterial isolates from patients.

Conventional AST is performed either in liquid culture media as in broth dilution on a 96-well plate format or on agar plates including disk diffusion or gradient strip methods [1]. Guidelines for these methods of determining MIC values are published by the Clinical and Laboratory Standards Institute (CLSI) [2]. These standardized tests are reliable but are labor-intensive and require long incubation times (typically 16–20 h) until the MIC can be determined by visual inspection of culture turbidity or colony growth.

Automated AST instruments (such as VITEK^®^ 2 and MicroScan WalkAway^®^) based on sensitive optical measurements have become commercially available. These instruments can accurately generate multiple test results in 3.5–16 h [1]. Although these instruments produce very quick and accurate results, their adoption by health care providers has been limited by their costs. In addition to initial instrument expenses, the high maintenance cost including purchasing AST cards required for each test is much greater than that of conventional AST methods, which is often prohibitive to low-resource healthcare systems servicing populations at even higher risk of infectious diseases. Therefore, a method that combines the availability of conventional AST protocols with the speed of automated instruments is highly desired.

A microfluidic approach, which allows for miniaturization of conventional biological assays, can be a viable solution to this technological problem. Wong and coworkers [3] have demonstrated that the inherently large surface-to-volume ratio of microfluidic devices made of a gas-permeable material of poly(dimethylsiloxane) (PDMS) allows for rapid growth of bacteria without the need for external agitation or oxygenation. Similarly, culture platforms such as agarose-filled microchannels [4,5], agarose microparticles [6], and nanoliter-scale droplets [7] were demonstrated to be compatible with AST.

Various methods for detecting bacterial growth have been utilized. Mach *et al.* [8] developed an RNA-specific electrochemical biosensor to determine the concentration of bacteria in solution and performed AST on clinical urine samples. The pH change of culture media during cell growth has also been used. Wu and coworkers [9] constructed a device made of chitosan hydrogel that is sensitive to pH changes. Weibel and coworkers [10] developed a self-loading microfluidic device based on pH-dependent colorimetric dyes allowing for visual examination. Another notable study by Sinn *et al.* [11] reported using asynchronous magnetic bead rotation to monitor bacterial growth starting from a single cell or low-density cultures. Image analysis software was used to measure the decrease in rotational velocity of the magnet as its surrounding medium became more viscous due to bacterial growth. Mohan *et al.* [12] constructed an integrated and multiplexed device that is capable of mixing reagents for performing AST based on a fluorescence signal from genetically engineered *E. coli* expressing green fluorescent protein, limiting its applicability to other types of bacteria. However, these designs incorporate more complicated fabrication and operation methods than those of conventional AST tests. In addition, several of these devices have developed their own metric of reporting MIC-like values.

We propose here a simple microfluidic device to perform rapid AST and determine MIC that does not depart from the traditional strategy and criteria set for the standard tests by CLSI. We miniaturize AST by combining a concentration gradient generator with cell culture chambers. The microfluidic channel network generates a serial dilution series of antibiotics mimicking that of the standard broth microdilution method. The microchambers for cell culture allow for monitoring bacterial response to antibiotic treatment via microscopic inspection. We demonstrate that MIC values consistent with those from 96-well plate experiments can be obtained in 3 h by using a model strain, *E. coli* ATCC 25922, treated with ampicillin and streptomycin.

There have been recent publications reporting similar approaches using miniaturized and/or parallelized fluidic channels and microscopic monitoring of cell growth. Wong *et al.* [13] used very thin microchannels to capture single bacteria electrokinetically and was able to obtain an antibiotic susceptibility profile in an hour. Suzuki *et al.* [14] showed that 360-nL microchambers combined with confocal reflection microscopy can be used to determine MICs within 12 h. A larger fluidic platform made of parallel 32 polyester channels was used for accurate methicillin-resistant *Staphylococcus aureus* (MRSA) phenotype testing of multiple bacteria species with automated microscopy [15,16,17]. Kwon *et al.* [18] demonstrated AST within 4 h at a higher throughput using 96-well plates filled with bacteria containing agarose combined with automated image acquisition and processing.

We believe that our platform is one of the simplest microfluidic devices available for performing rapid AST. It performs AST in 3 h, it is inexpensive, and it is easy to fabricate. In addition, we have developed a straightforward method of introducing the bacterial solution into the chip via a manually operated syringe injection. Our approach could be a valuable addition to the field of on-chip AST as it emulates traditional AST and reports rapid MIC at rates comparable to those of other miniaturized devices and automated AST instruments.

## 2. Results and Discussion

### 2.1. Microchip Design

**Figure 1:**
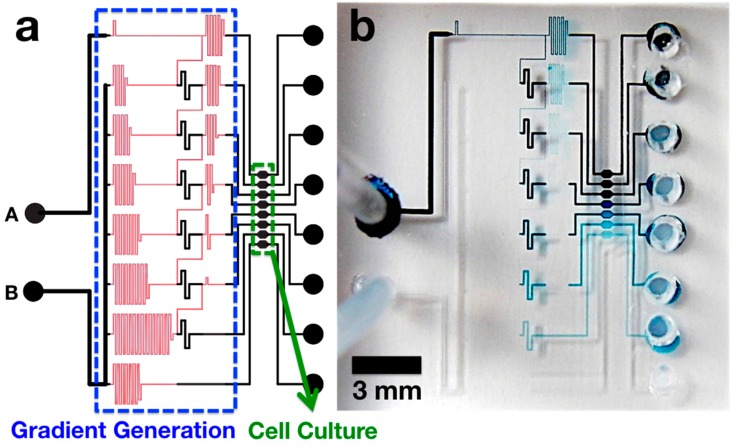
(**a**) An illustration of microchip design: black and red channels represent 149-μm and 4-μm tall features, respectively; (**b**) A photograph of the fabricated PDMS chip. Blue food dye solution and water were injected to upper (**A**) and lower (**B**) inlet ports, respectively.

Figure 1 shows the design of the AST microchip. We adapted the microfluidic network structure for generating monotonic concentration profiles as proposed by Hattori, Sugiura, and Kanamori [19]. The gradient generation region was composed of 149-μm-deep mixing channels (low fluid resistance) that are sandwiched between 4-μm-deep channels for resistance adjustment (high fluid resistance) and interconnected by 4-μm-deep vertical channels. The length of each channel element was tuned to achieve a two-fold serial dilution series in the top 7 channels. The bottom channel serves as a control without any antibiotics. Then, the channels are connected to 30-nL cell culture chambers. When an aqueous solution of food dye was injected with pressure through the input ports, an obvious series of decreasing concentration was readily observed by eye, as shown in Figure 1b.

### 2.2. Characterization of Generated Concentration Gradient

**Figure 2:**
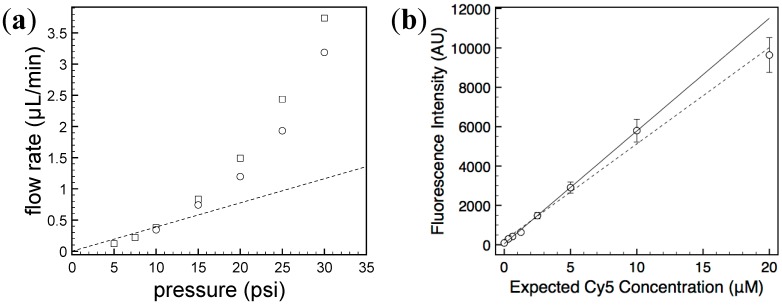
(**a**) Flow rates achieved by different injection methods: pressure controller (□) and syringe compression (○). The microchannel used for this measurement had a dimension of 24 mm × 70 μm × 7 μm (*L* × *W* × *H*). The dashed line stands for the linear relationship between flow rate (*Q*) and injection pressure (Δ*P*) expected for a rectangular channel without any deformation: ∆*P* = 12 *μQLH*^−3^*W*^−1^, where *μ* is the fluid viscosity of water (assumed to be 0.001 Pa s). The fluidic resistance for the tested channel is 29 psi min μL^−1^. The deviation from the linearity results from deformation of the PDMS channel [20]; (**b**) Fluorescence intensity measured from each culture chamber after injecting 20 μM Cy5 solution. The error bars stand for standard deviations calculated from 100 × 100 pixel areas. The linear regression analysis of all 8 data points resulted in *y* = 489.22 *x* + 232.17 (*R*^2^ = 0.991; dashed line). The same analysis without the point at the highest concentration produced *y* = 572.71 *x* + 51.44 (*R*^2^ = 0.999; solid line).

First, manual pressure injection using a syringe (summarized in Supplementary Figure S1) was tested and compared with injection by a pressure controller. The resulting flow rates were comparable between the two methods as shown in Figure 2a, demonstrating that compression injection based on simple syringe attachment can be used to operate our microdevice without the need for a compressed air source or pressure regulator.

To confirm whether the generated concentration series was truly exponentially decreasing with a factor of two, a Cy5 solution was injected to the chip and laser-induced fluorescence signals from the culture chambers were recorded with an EMCCD camera. The average signal intensities from the acquired fluorescence images showed an excellent linear relationship with expected fluorophore concentration (Figure 2b) (*R*^2^ value of 0.991). A better quality of fitting (*R*^2^ = 0.999) was obtained when the data point having the highest fluorescence signal was excluded, suggesting the possibility of imperfect chip design or more pronounced nonlinearity of fluorescence quantum yield at a higher concentration. Nonetheless, the error in the generated concentration profile is less than 10% for all data points, which is sufficient for executing the AST protocol.

### 2.3. MIC Determination Using the Standard Broth Microdilution (SBM) Method

**Figure 3:**
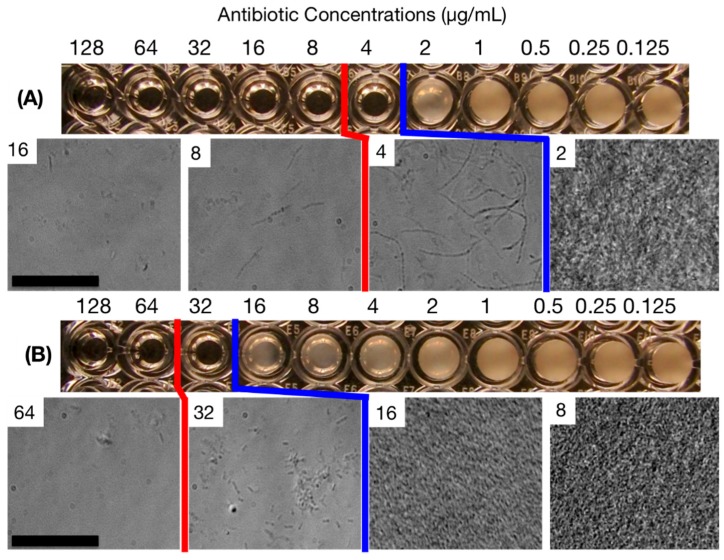
Off-chip MIC determination of *E. coli* based on the SBM method (22 h of incubation): (**A**) ampicillin and (**B**) streptomycin. MIC determination criteria by visual and microscopic inspections are shown in blue and red lines, respectively. The numbers in the micrographs represent the concentrations of antibiotic in μg/mL. Scale bars = 50 μm.

*E. coli* ATCC 25922 cells were exposed to two types of antibiotics, ampicillin and streptomycin, which work through different mechanisms. Ampicillin inhibits the transpeptidase enzyme that is necessary for cell wall synthesis. Streptomycin binds to the bacterial ribosome and inhibits protein synthesis. When the SBM method in a 96-well plate format was used, we observed culture turbidity for 2 μg/mL and 16 μg/mL for ampicillin and streptomycin, and thus MIC values of 4 μg/mL and 32 μg/mL, respectively (Figure 3). However, with microscopic inspection, we observed slow but clear growth of cell populations at these concentrations. At 4 and 8 μg/mL ampicillin, the cells develop an uncharacteristic elongated shape indicating that the cell division and/or cell wall formation is frustrated. At higher concentrations, the cell counts decreased from the initial values, reflecting the bactericidal property of ampicillin via induced cell lysis. With streptomycin, such a phenotypic change was not observed, but there existed a non-negligible cell growth at 32 μg/mL. Therefore, we determined “microscopic” MIC values to be 8 and 64 μg/mL for ampicillin and streptomycin, respectively.

### 2.4. MIC Determination Using Microchip AST 

**Figure 4:**
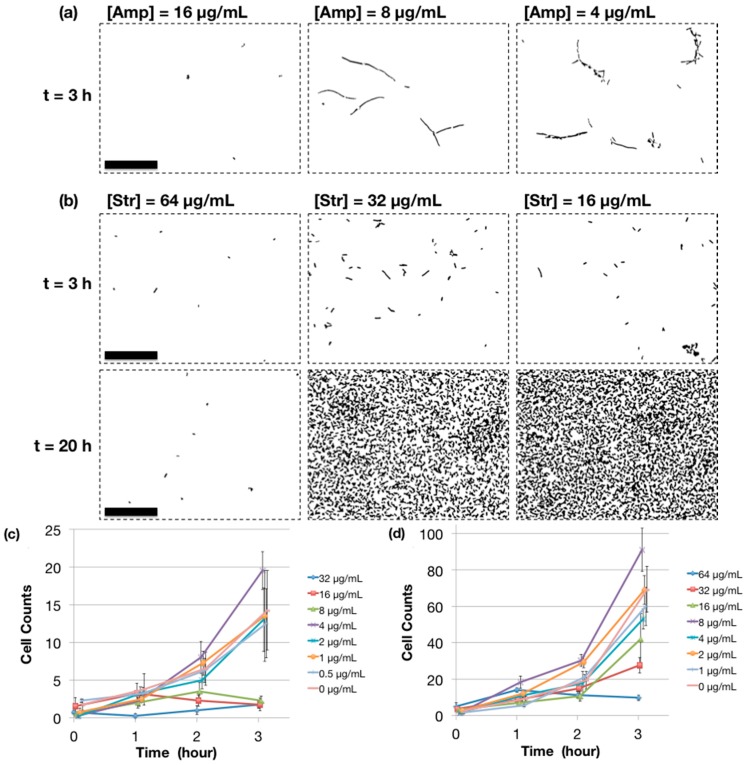
On-chip MIC determination based on antibiotic gradient generation. The images are selected to be representative of the cell growth pattern observed in the micro culture chamber and processed with the ImageJ software as detailed in the Experimental Section. (**a**,**c**) ampicillin (Amp)-treated *E. coli* cells and (**b**,**d**) streptomycin (Str)-treated *E. coli*. The average cell counts (in the 100 μm × 75 μm region of interests) were calculated to determine the breakpoints for MIC, which are 5 cells and 16 cells for Amp and Str, respectively. Scale bars = 50 μm.

We tested the performance of our microchip for AST by injecting a bacterial suspension of the same cell density level as the SBM protocol and monitoring cell growth with microscopic imaging (Figure 4). After 2 h of incubation, cell growth patterns within the culture chambers started to show difference depending on the antibiotic concentrations (Supplementary Figure S2). For ampicillin, at the concentration of 8 μg/mL, the elongated species were observed but no significant changes in cell counts. At lower antibiotic concentrations, *E. coli* cells start to form conglomerates, showing that the supplied drug can no longer suppress active growth of bacteria. At 3 h of incubation, the trend becomes clearer and the determination of MIC (8 μg/mL) is evident, which agrees well with the standard 96-well plate result (Figure 4a,c). It is interesting to note that, at 4 μg/mL ampicillin, the elongated phenotype is not so pronounced as that of 96-well plate data. We speculate that either the gas exchange property of the 30-nL microchamber, which is better than non-agitated 96-well plate, or limited availability of total ampicillin amounts arising from the small volume played a role here. For streptomycin, we observed bacterial proliferation starting from 32 μg/mL, thereby agreeing with the microscopic MIC value of 64 μg/mL determined from the SBM method that we performed (Figure 4b,d).

## 3. Experimental Section

### 3.1. Bacterial Culture

*Escherichia coli* ATCC 25922 (*E. coli*) was obtained from ATCC (Manassas, VA, USA). This gram-negative bacterial species is recommended by the Clinical Laboratory Standards Institute (CLSI) as the quality control strain for AST. Mueller-Hinton Broth (MHB) was purchased from Thermo Scientific (R112474; Waltham, MA, USA). A fresh *E. coli* culture was prepared for each experiment by inoculating 1 mL of MHB with 2 μL stock solution and incubating at 37 °C overnight. We estimated cell density by measuring absorbance at 600 nm with a spectrophotometer (DU-520, Beckman Coulter, Brea, CA, USA; Figure S3 in Supplementary). The actively growing culture was diluted with MHB to a desired cell density level for further experiments.

### 3.2. MIC Determination by SBM Method

According to CLSI-recommended guidelines (M7-A7) [21], a panel of liquid culture with varying concentrations of antibiotics was prepared in a 96-well plate (Microtest 96, Beckton Dickinson, Franklin Lake, NJ, USA). Briefly, a series of two-fold dilutions of antibiotic drug was prepared over a range of concentrations from 128 μg/mL to 0.125 μg/mL. Ampicillin (cat. # A0166) and streptomycin (cat. # S9137) were obtained from Sigma-Aldrich (St. Louis, MO, USA). Each well contained 150 μL of cell culture media and the initial cell density was set to 5.0 × 10^5^ cells/mL. After overnight (18–20 h) incubation at 37 °C in a humid chamber, the plate was visually inspected to determine which wells turned turbid, and images were taken through a microscope.

### 3.3. Microchip Fabrication

PDMS chips were fabricated using soft lithography as previously reported with minor modifications [22,23]. Briefly, the master molds for PDMS casting were made on a silicon wafer with SU-8 photoresist (MicroChem, Westborough, MA, USA) via standard photolithography at the Stanford Nanofabrication Facility. Cell culture chambers and main channels were 149-μm high and the fluidic network structures for creating concentration gradient were 4-μm high. The volume of each culture chamber was about 30 nL. PDMS prepolymers (RTV615; Momentive, Waterford, NY, USA) were mixed at a 10:1 ratio (Part A:Part B), poured onto the master, degassed and cured at 80 °C for 2 h. After cooling, the 4-mm-thick PDMS slab was peeled off and holes were punched. For final bonding, the surface of PDMS piece was activated using a plasma cleaner (PDC-32G, Harrick Plasma, Ithaca, NY, USA). The air pressure was held at 5 mTorr for 15 s and then increased quickly to 100 Torr. Such a pressure cycle was repeated for 6 times. Immediately after plasma treatment, the channel side of the PDMS slab was placed with a gentle pressure onto a precleaned glass slide (25 × 75 × 1 mm; Fisher Scientific, Hampton, NH, USA).

### 3.4. Fluorescence Intensity Measurements

To check the performance of the fabricated chip in terms of monotonic serial dilution capability, an aqueous solution of Cy5 dye (20 μM) (PA25001; GE Healthcare, Pittsburgh, PA, USA) was introduced into the upper and the lower input ports, respectively. The Cy5 solution was supplemented with 0.1% (*v*/*v*) sodium dodecyl sulfate (BP1311-200; Fisher Scientific, Waltham, MA, USA) to prevent adsorption of dye molecules to the channel wall. Each chamber was illuminated with an expanded beam of a 638 nm diode laser (RCL-025-638, CrystaLaser, Reno, NV, USA); the resulting fluorescence was collected through a dichroic mirror (400-535-635TBDR; Omega Optical, Brattleboro, VT, USA) and a bandpass filter (HQ675/50m; Chroma Technology, Bellows Falls, VT, USA) and imaged using an EMCCD camera (iXon DU897BV, Andor Technology, Belfast, NIR, UK); the EM gain level was 8 and the exposure time 100 ms. For data analysis, the signals from a 100 × 100 pixel area centered around the peak intensity point were averaged using the manufacturer’s software (Andor SOLIS 4.11).

### 3.5. Compression Injection

Twenty microliters of 5.0 × 10^5^ cells/mL *E. coli* suspensions in MHB (supplemented with 0.1% (*v*/*v*) Tween 20) with 32 and 0 μg/mL ampicillin were introduced to the upper and the lower input ports, respectively, and the solutions were injected into the chip by applying air pressure at 20 psi for 5 min. Tween 20 (P5927; Sigma-Aldrich, St. Louis, MO, USA) was added to prevent cell agglomeration and sticking to the microchannel walls. Addition of 0.1% (*v*/*v*) Tween 20 did not affect cell growth in MHB (data not shown). Sixty-milliliter syringes (309653; Becton Dickinson, Franklin Lakes, NJ, USA) filled with air were used to apply the pressure. A syringe was connected to each input port with Tygon^®^ tubing. The syringes were pressed down simultaneously to 25.4 mL from 60 mL of air volume in order to exert 20 psi of pressure on the bacterial suspensions at the input ports (Supplementary Figure S1). Twenty psi was found to be the optimal pressure in order to fill the microfluidic chip rapidly yet preventing the formation of air bubbles or deformation of the chip. More detailed information about the injection process is presented in the supplementary information.

### 3.6. MIC Determination by Microchip AST

Once the microfluidic chip was filled, it was incubated at 37 °C in a humid chamber (TS Auto Flow, NuAire, MN, USA) for 3 h with sealed inlet and outlet ports to prevent evaporation. The bacterial population in each culture chamber was observed using a phase contrast microscope (Diaphot 300; Nikon, Melville, NY, USA) and a 40× magnification lens. Microscopic images of predetermined locations in the incubation chambers were obtained at one-hour intervals using a CCD camera (Infinity2-1M, Lumenera, Ottawa, ON, Canada). The MIC determination was repeated at least three times to confirm reproducibility.

### 3.7. Image Processing by ImageJ Software

Acquired microscopic images were processed further with the freely available ImageJ software (Rasband, W.S., N.I.H., Bethesda, MD, USA, http://imagej.nih.gov/ij/, version: 2.0.0-rc-34/1.50a) to remove optical artifacts in the following sequences: (a) apply Fast Fourier Transform (FFT) bandpass filter with an upper bound of 25 pixels and a lower bound of four pixels with the Autoscale option ON; (b) threshold with the IsoData scheme (and adjust the range if necessary); (c) use “Analyze Particles” function to identify cells only using the parameters: Size = 100–Infinity (pixel^2^), Circularity = 0.00–0.85, and Show = Masks. An example of processed images can be found in the Supplementary Data (Figure S4).

## 4. Conclusions

Our data demonstrate that a miniaturized version of AST analysis can be achieved utilizing a microchannel network structure for creating a dilution series of antibiotic concentrations and providing cell culture chambers for efficient gas exchange. Microchip AST can determine MIC values as early as 3 h via direct microscopic inspection of cell growth, which is as rapid as commercial automated AST systems. Our scheme requires only 10–20 μL of antibiotic stock solutions (diluted to the highest concentration in the test range), an incubation chamber, and a simple microscope for cell growth monitoring. Therefore, this kind of microchip can be manufactured and sent for usage to any laboratory equipped with minimal resources for clinical microbiology analyses. We suggest that this will reduce AST cost and expand the availability of the technology significantly. The microfluidic channels can be adjusted to generate a linear scale or an arbitrary concentration profile. This feature might be useful for studying pharmacokinetics and bacterial response to antibiotics. The chip design can also be extended to handle multiple bacterial species and/or drug types to ensure uniform experimental conditions for comparison and to increase the data throughput. However, it should be noted that bacterial species with different sizes, motilities or growth rates may be more challenging for rapid AST by affecting imaging accuracy and/or growth pattern on our platform, which warrants a further study to generalize this approach to other disease-related bacteria, such as *Pseudomonas aeruginosa*, *Staphylococcus aureus*, and *Enterococcus faecalis*.

## Supplementary Files

Supplementary File 1
