# Supplementary material for: Miniaturized Antimicrobial Susceptibility Test by Combining Concentration Gradient Generation and Rapid Cell Culturing
Source: Antibiotics (Basel). 2015 Oct 29;4(4):455–66. doi: 10.3390/antibiotics4040455 (PMC4790307; doi:10.3390/antibiotics4040455)
Supplement: Supplementary File 1 [file antibiotics-04-00455-s001.pdf]

Supplementary Materials

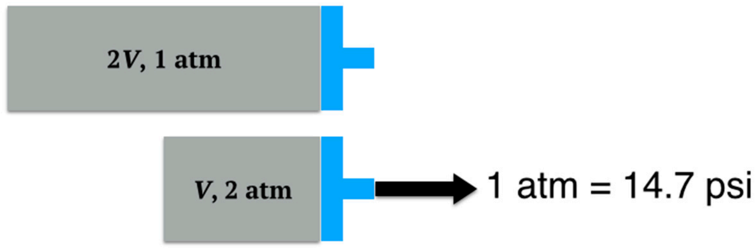

Assuming the air pressure to be 1 atm (= 14.7 psi), if 60 mL of air in the syringe is compressed to 30 mL, then the additional pressure onto the liquid surface will be 14.7 psi.

The compression ratio to achieve 10, 20 or 30 psi injection pressure can be calculated as follows:

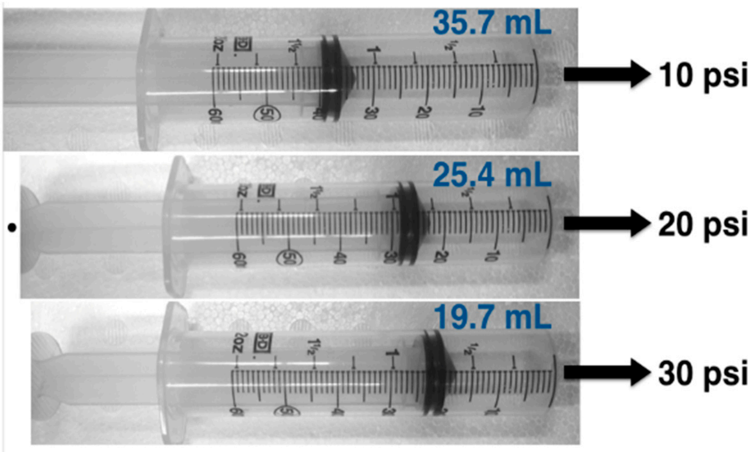

For x psi extra pressure, final pressure should be (x + 14.7) psi. Using Boyle's law (PV = constant),

$$(14.7)\ (V_0) = (x + 14.7)\ (V_1)$$

$$V_1 / V_0 = 14.7 / (x + 14.7)$$

For V_0 = 60 mL,

x = 10 psi, V_1 = 35.7 mL

x = 20 psi, V_1 = 25.4 mL

x = 30 psi, V_1 = 19.7 mL

Figure S1. Compression injection via 60-mL syringe.

(**a**) Ampicillin

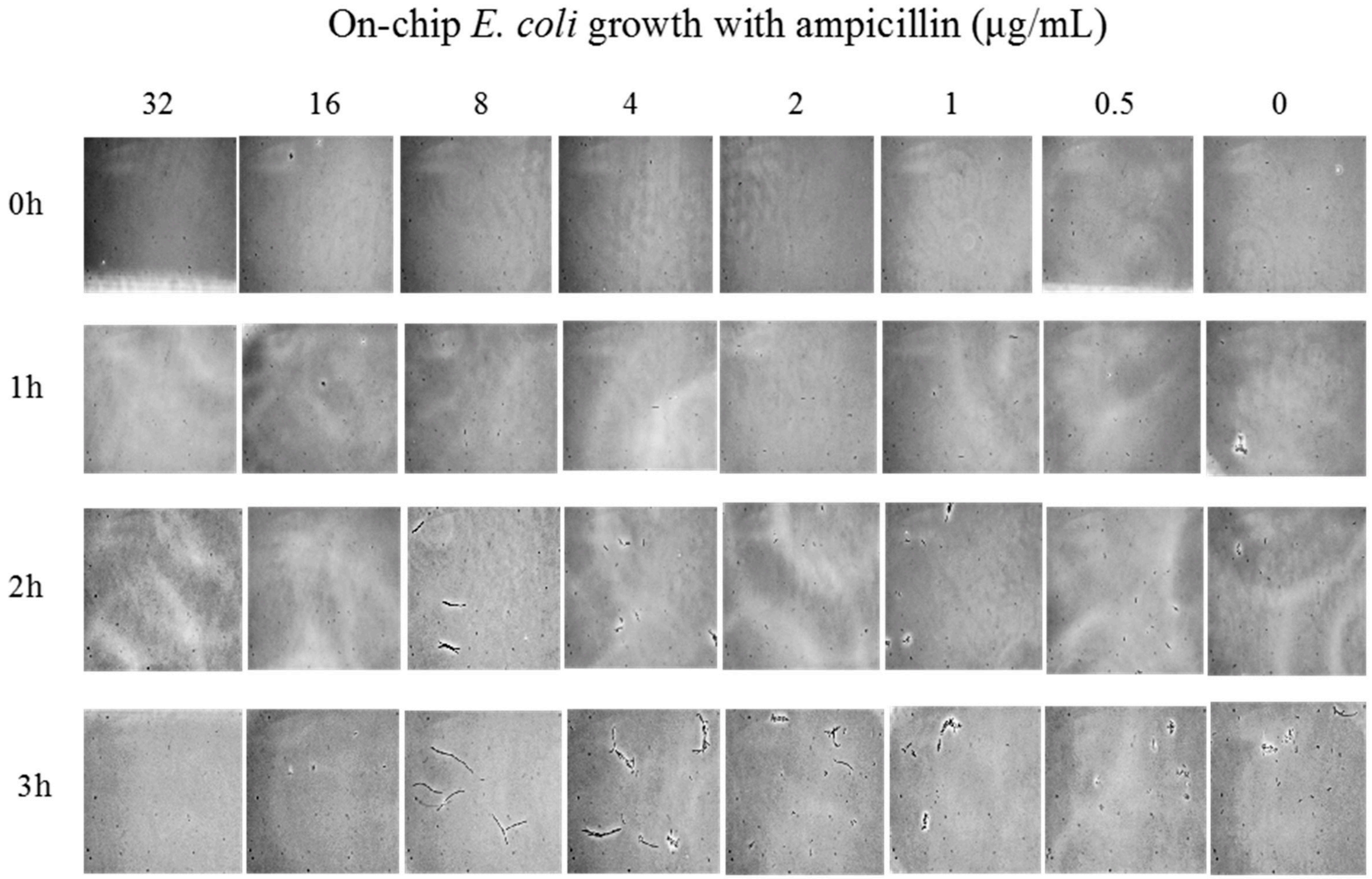

Figure S2. *Cont.*

(**b**) Streptomycin

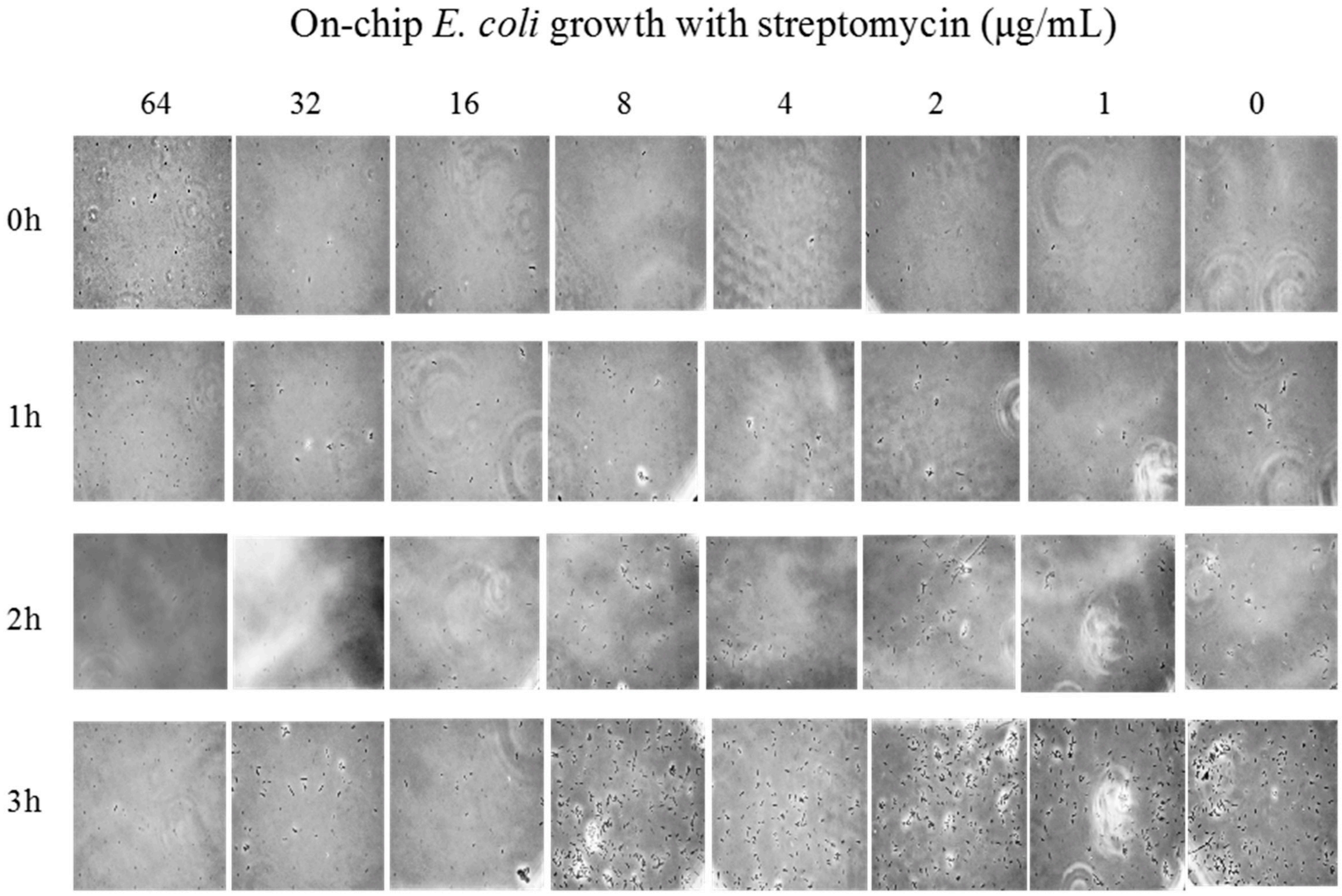

Figure S2. Microscope images of *E. coli* growth within microchambers.

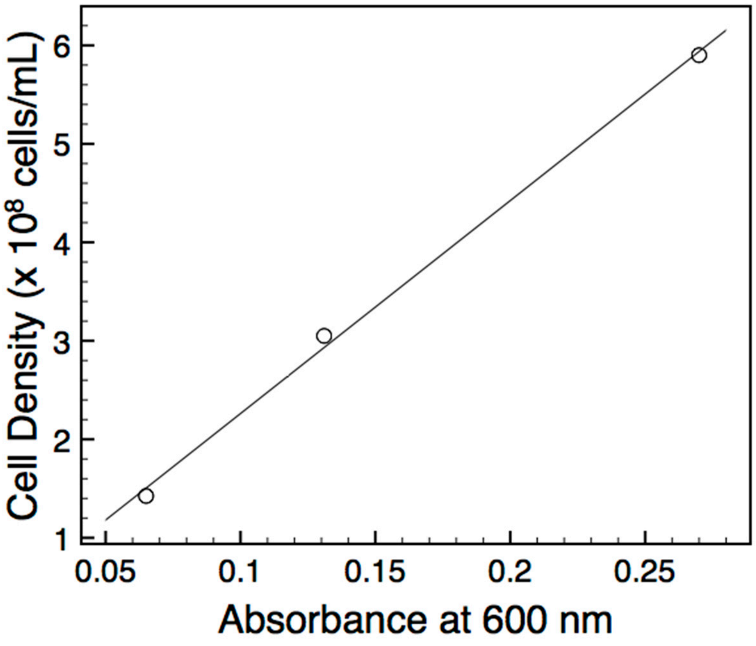

Figure S3. Relationship between absorbance at 600 nm and cell density. Cell density for each sample was determined using a hemocytometer. Linear regression analysis resulted in: $y = 21.625\,x + 0.099$ ($R^2 = 0.998$) as shown by the line.

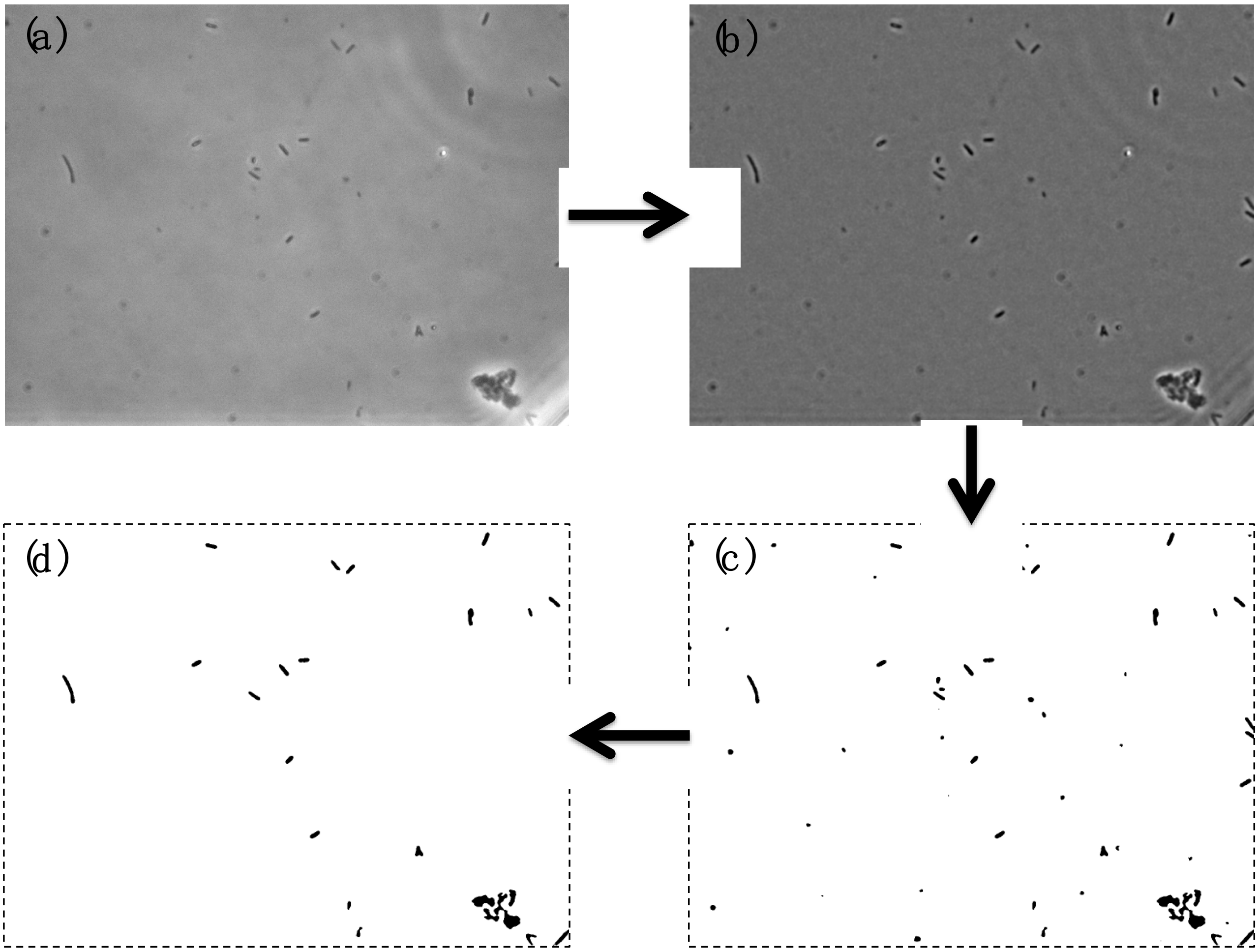

Figure S4. Image Processing for Microscopic Images of Cells: (**a**) raw image, (**b**) FFT bandpass filtered image, (**c**) IsoData thresholded image and (**d**) Size/Circularity-filtered image.

© 2015 by the authors; licensee MDPI, Basel, Switzerland. This article is an open access article distributed under the terms and conditions of the Creative Commons Attribution license (http://creativecommons.org/licenses/by/4.0/).
